# Twist2 contributes to skin regeneration and hair follicle formation in mouse fetuses

**DOI:** 10.1038/s41598-024-60684-5

**Published:** 2024-05-13

**Authors:** Kento Takaya, Ayano Sunohara, Shigeki Sakai, Noriko Aramaki-Hattori, Keisuke Okabe, Kazuo Kishi

**Affiliations:** Department of Plastic and Reconstructive Surgery, Keio University School of Medicine, 35 Shinanomachi, Shinjuku-Ku, Tokyo, 160-8582 Japan

**Keywords:** Twist2, Scar, Skin regeneration, Hair follicle formation, Wound healing, Disease model, Morphogenesis, Pattern formation, Mechanisms of disease

## Abstract

Unlike adult mammalian wounds, early embryonic mouse skin wounds completely regenerate and heal without scars. Analysis of the underlying molecular mechanism will provide insights into scarless wound healing. Twist2 is an important regulator of hair follicle formation and biological patterning; however, it is unclear whether it plays a role in skin or skin appendage regeneration. Here, we aimed to elucidate Twist2 expression and its role in fetal wound healing. ICR mouse fetuses were surgically wounded on embryonic day 13 (E13), E15, and E17, and Twist2 expression in tissue samples from these fetuses was evaluated via in situ hybridization, immunohistochemistry, and reverse transcription-quantitative polymerase chain reaction. Twist2 expression was upregulated in the dermis of E13 wound margins but downregulated in E15 and E17 wounds. Twist2 knockdown on E13 left visible marks at the wound site, inhibited regeneration, and resulted in defective follicle formation. Twist2-knockdown dermal fibroblasts lacked the ability to undifferentiate. Furthermore, Twist2 hetero knockout mice (Twist + /-) formed visible scars, even on E13, when all skin structures should regenerate. Thus, Twist2 expression correlated with skin texture formation and hair follicle defects in late mouse embryos. These findings may help develop a therapeutic strategy to reduce scarring and promote hair follicle regeneration.

## Introduction

The economic and social consequences of scarring are significant. More than 100 million patients acquire scars each year, some of which cause considerable functional or psychosocial morbidity^1,2^. Therefore, the development of effective therapies for scarless wound healing has a major impact and has gained considerable attention.

Wound healing is a complex process that usually occurs in the postnatal period through the formation of scar tissue, and regenerative healing in adult animals is limited to liver and bone^3,4^. In contrast, the skin wounds of fetuses are known to heal without scars by regenerating normal skin structures, including the recovery of cutaneous appendages and neurovasculature up to a specific developmental stage^5,6^. Scar tissue is characterized by (1) fibrosis of the dermis, (2) loss of skin texture, (3) loss of skin appendages, and (4) changes in color tone. Therefore, to determine whether a wound has regenerated, the morphology of the scar (excluding color tone) should be evaluated from two perspectives: skin texture and dermal structure^7,8^. In a model of wound healing in mammalian fetuses, an early regenerative healing period is followed by a period in which the wound heals with an extracellular matrix (ECM) that is indistinguishable from intact tissue but fails to regenerate dermal appendages, leaving a visible mark^9^. Thereafter, late in development, the wound changes to an adult-like phenotype with the wound healing due to excess collagen in the ECM, loss of dermal appendages, and flattening of the epidermis, classified as a scar. Until now, this change was thought to occur progressively. However, using our proprietary mouse fetal wound model, we found a critical point where complete regeneration without visible scarring occurs until embryonic day 13 (E13) and regeneration of dermal structures occurs until E16, after which, the wound becomes similar to the adult animal type^10,11^. A deeper understanding of the basic mechanisms of fetal wound healing will allow us to identify therapeutic targets that can minimize scar formation. In particular, observing the factors that change before and after the critical point may be important for promoting post-wound skin regeneration. Skin appendages, such as bird feathers, mammalian hair, and zebrafish scales, have long been considered essential for interactions between the epidermis and dermis^12^. Several signaling pathways and components, such as Wnt, fibroblast growth factor (FGF), and Sonic hedgehog (SHH), and transcription factors, such as Twist, have been shown to be essential in regulating feather and hair formation and patterning^13–15^.

Twist1 and Twist2 (also called Dermo1) are highly conserved basic helix-loop-helix transcription factors that are structurally conserved, containing basic amino acids flanking two amphipathic alpha-helices separated by an interhelix loop^16^. Twist plays an important role in dorsoventral patterning and mesodermal incision in *Drosophila*^17,18^. In chicken, Twist2/Dermo1 is expressed in feather-forming cells, indicating its role in feather formation^19^. In mice, Twist2 knockout reportedly caused progressive growth retardation, possibly as a result of upregulated cytokine signaling, leading to thin skin and sparse distorted hair follicles before death^20^. Thus, while Twist2 signaling is involved in patterning and hair follicle formation, it is unclear whether these paralogs have a primary function in skin texture or skin appendage regeneration. However, as patterning has been reported to be important for epidermal texture and hair follicle formation^21^, we hypothesized that Twist2 is involved in skin regeneration after wounding during early development.

In this study, we aimed to determine whether Twist2 plays a role in wound healing using a mouse model. As fetal mouse wounds epithelialize between 48 and 72 h after wounding^10^, we focused on wounds 24 h after wounding to observe the effect of Twist2 on the wound healing process. Next, to analyze the function of Twist2, we knocked it down using a small interfering RNA (siRNA) and examined its effect on wound healing. Furthermore, as Twist2 homozygous knockout mice are lethal and heterozygous knockout mice are viable and fertile, we observed the wound healing process in the fetuses of Twist + /- mice^17^. In addition, we investigated the relationship among dermal fibroblast function, hair follicle formation, and Twist2 expression. We investigated this relationship as previous studies have shown that dermal fibroblasts lose their ability to form hair follicles when cultured in normal two-dimensional (2D) culture, but when cell aggregates are formed in nonadherent culture dishes, they regain their ability to form hair follicles, express undifferentiated markers such as Sox-2 and CD133, and acquire pluripotency^19^. We investigated the effects of Twist2 on wound regeneration/repair and hair follicle formation using our own fetal mouse wound healing model and a three-dimensional cell model.

## Results

### Twist2 expression is upregulated in the dermis of E13 wounds

**Figure 1:**
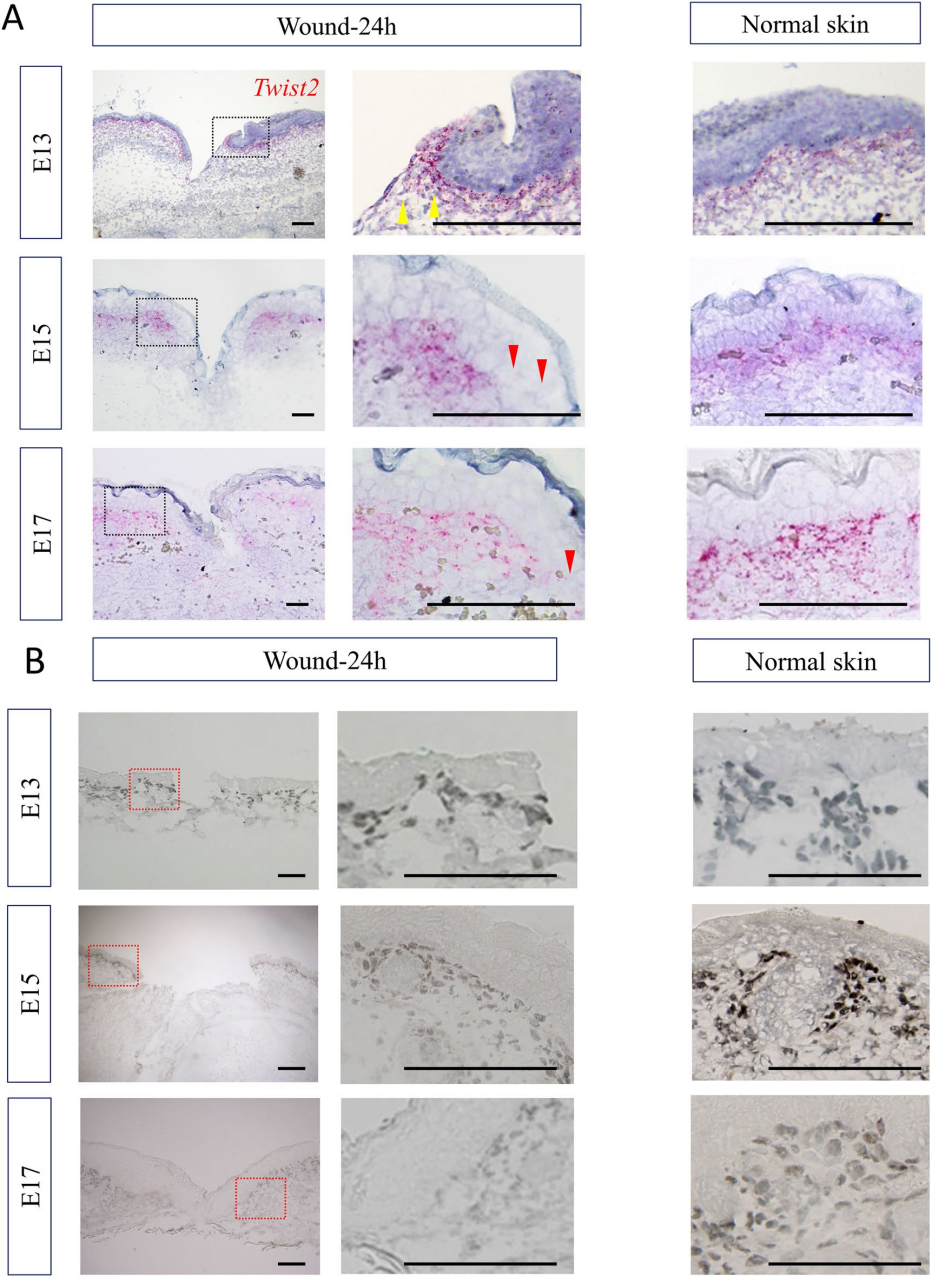

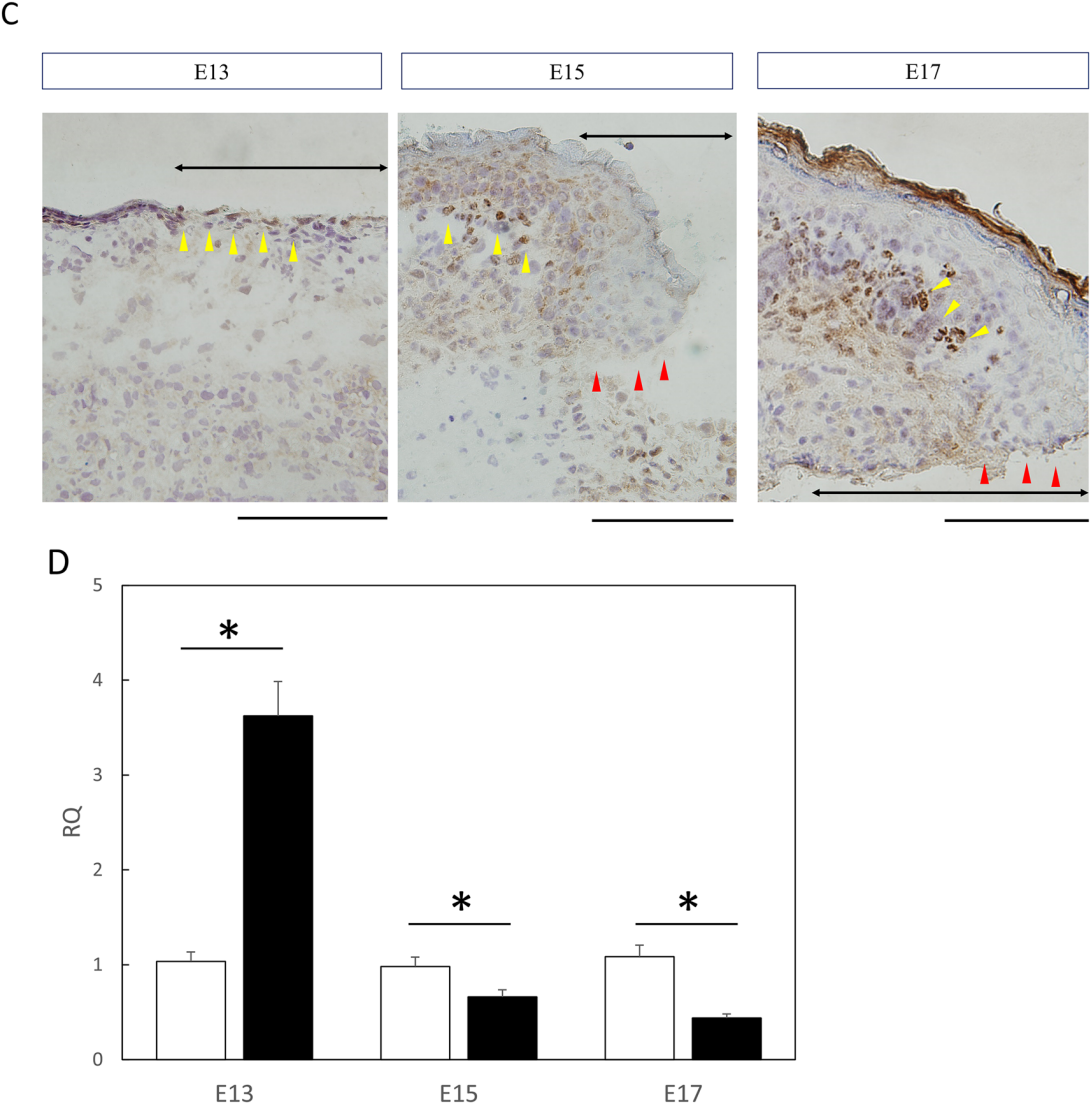
Twist2 expression during wound healing in fetal mouse embryos. (**A**) In situ hybridization analysis. Twist2 expression was upregulated in the dermis of the wound margin on E13 but downregulated from E15 onwards. Yellow arrows: upregulated area. Red arrows: downregulated area. Bar = 100 µm. (**B**) Immunohistochemical staining of Twist2. Bar = 100 µm. (**C**) Observation of Twist2 and nucleus location using immunohistochemical staining. Brown: Twist2, Blue: nucleus. Yellow triangles: areas where twist2 is observed in the nucleus. Red triangles: areas where Twist2 is not expressed. Black arrow; wound area. Bar = 100 µm. (**D**) qPCR analysis of Twist2 gene expression in wound and normal areas. RQ, relative quantification; qPCR, quantitative polymerase chain reaction. *P < 0.05. All experiments were repeated three times.

In situ hybridization was performed to observe the expression of *Twist2* during wound healing in embryonic development. The results showed that Twist2 mRNA was specifically expressed in the dermis, and its expression was upregulated as it approached the epidermis. In relation to the wound, Twist2 mRNA expression was high at the wound margin on E13 and tended to decrease as it approached the wound margin on E15 and E17 (Fig. 1A).

In immunostaining, Twist2 protein was specifically expressed in the dermis, and its expression was upregulated as it approached the epidermis. In addition, the expression of Twist2 protein on E15 and E17 decreased around the wound site compared with that at the normal site, whereas that on E13 tended to increase at the wound site compared with that at the normal site (Fig. 1B).

To observe the location of Twist2 and nuclei, nuclear counterstaining was performed along with immunostaining for Twist2. We found that Twist2 was expressed in the dermis of E13 wound along with the nuclei. Contrarily, on E15 and E17, Twist2 was found to be expressed in the non-wound dermis along with the nuclei, indicating that its expression decreased in the wound (Fig. 1C).

When mRNA was extracted from only dermis of the wound via laser microdissection (LMD) and analyzed using real-time polymerase chain reaction (PCR), the results revealed that Twist2 expression significantly increased in the wound compared with that in the normal skin on E13 (P = 0.02). In contrast, the expression on E15 (P = 0.03) and E17 (P = 0.036) decreased in the wound area compared with that in the normal skin (Fig. 1D). Thus, Twist2 expression is upregulated in fully regenerating E13 wounds and downregulated in later developmental stages, suggesting its involvement in the E13-type wound healing phenotype.

### Knockdown of Twist2 inhibits mouse fetal skin regeneration

**Figure 2:**
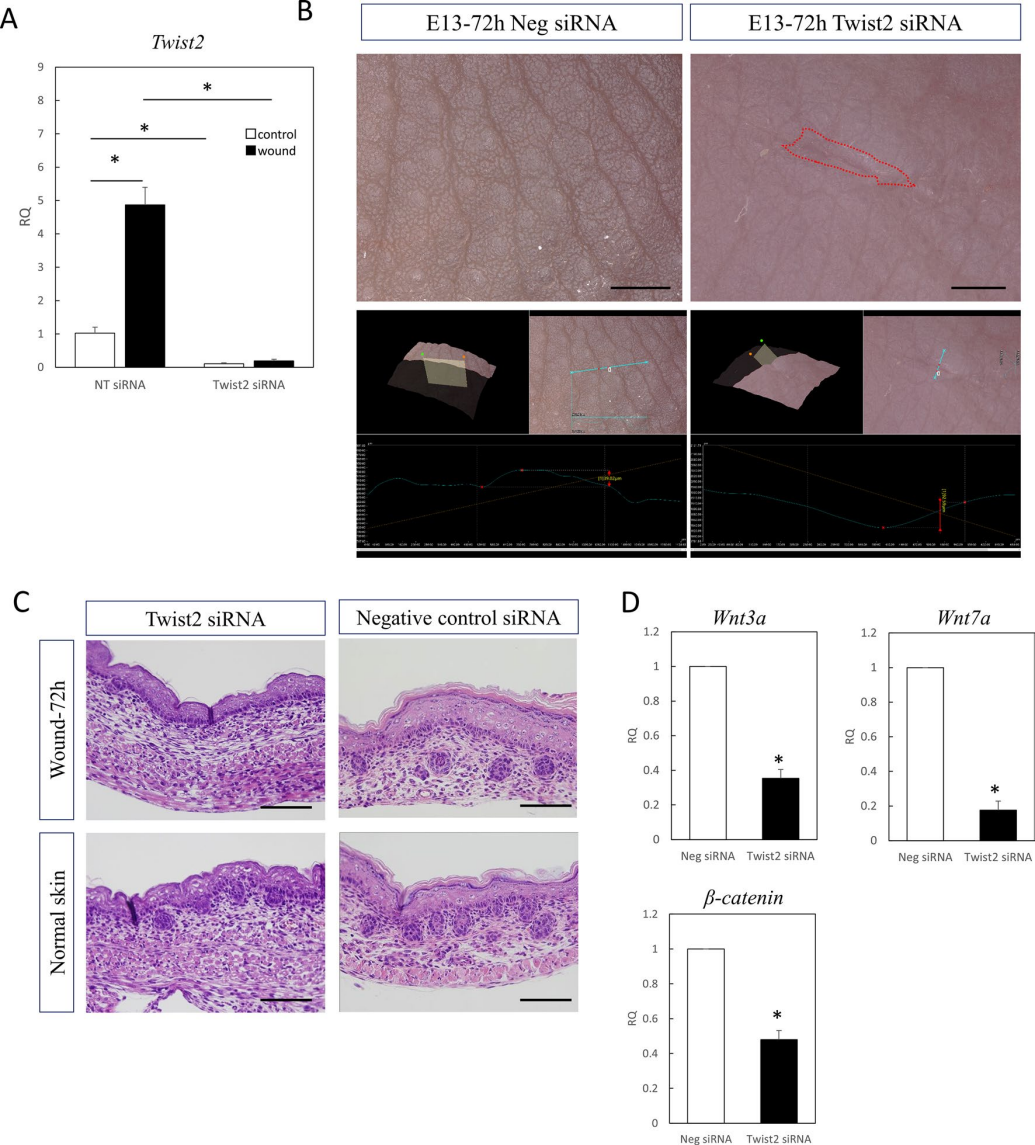
Knockdown of Twist2 expression in vivo using siRNA and its effect on wound healing. (**A**) Knockdown of Twist2 expression on E13. RQ, relative quantification. *P < 0.05. (**B**) Effect of Twist2 knockdown on wound healing. Bar = 1 mm. Red dotted line: visible mark of wound. (**C**) Hematoxylin and eosin staining of wound tissue after Twist2 knockdown. No follicle formation was observed in the Twist2-knockdown wound. (**D**) qPCR analysis of hair induction-related gene expression in wound areas. RQ, relative quantification; qPCR, quantitative polymerase chain reaction. *P < 0.05. Neg siRNA, negative control siRNA. Bar = 100 µm.

To evaluate whether Twist2 is required for fully regenerative E13-type wound healing, we knocked down Twist2 in the skin by injecting siRNA into the amniotic fluid. Mice analyzed 72 h after Twist2 siRNA administration during surgery showed a significant knockdown of Twist2 expression in both wounds and normal skin (control, P = 0.027; wound, P = 0.025). In addition, a significant knockdown of Twist2 expression was observed in both control and normal skin (control, P = 0.027; wound, P = 0.019). Conversely, in the group treated with negative control siRNA, Twist2 expression was significantly upregulated in the wound (P = 0.03; Fig. 2A). This finding confirmed the successful knockdown of Twist2 in fetal mouse skin via Twist2 siRNA administration.

In the morphological observation of wounds using a stereo microscope, the wounds were usually completely regenerated even in E13 mice, but treatment with Twist2 siRNA in amniotic fluid prevented skin regeneration, leaving visible marks with no regeneration of the skin structure (Fig. 2B).

Hematoxylin and eosin staining of tissue sections showed that hair follicle regeneration was lost in E13 wounds treated with Twist2 siRNA (Fig. 2C). In the negative control siRNA group, the skin structures, including hair follicles, completely regenerated, indicating that Twist2 is involved in skin regeneration.

The expression of *Wnt3a* (P = 0.00051), *Wnt7a* (P = 0.00042), and *β-catenin* (P = 0.0012), which encode Wnt/β-catenin signaling molecules associated with hair follicle induction, was found to be significantly downregulated in the wound skin of the Twist2 siRNA-treated group compared to the negative control siRNA-treated group (Fig. 2D).

### Wounds in Twist2 heterozygous knockout mice do not regenerate

The pattern of wound healing in Twist2-knockout mouse fetuses was observed. Crossing of Twist2 heterozygotes yielded homozygous (homo) and heterozygous (hetero) mutant offspring at the predicted Mendelian ratio.

**Figure 3:**
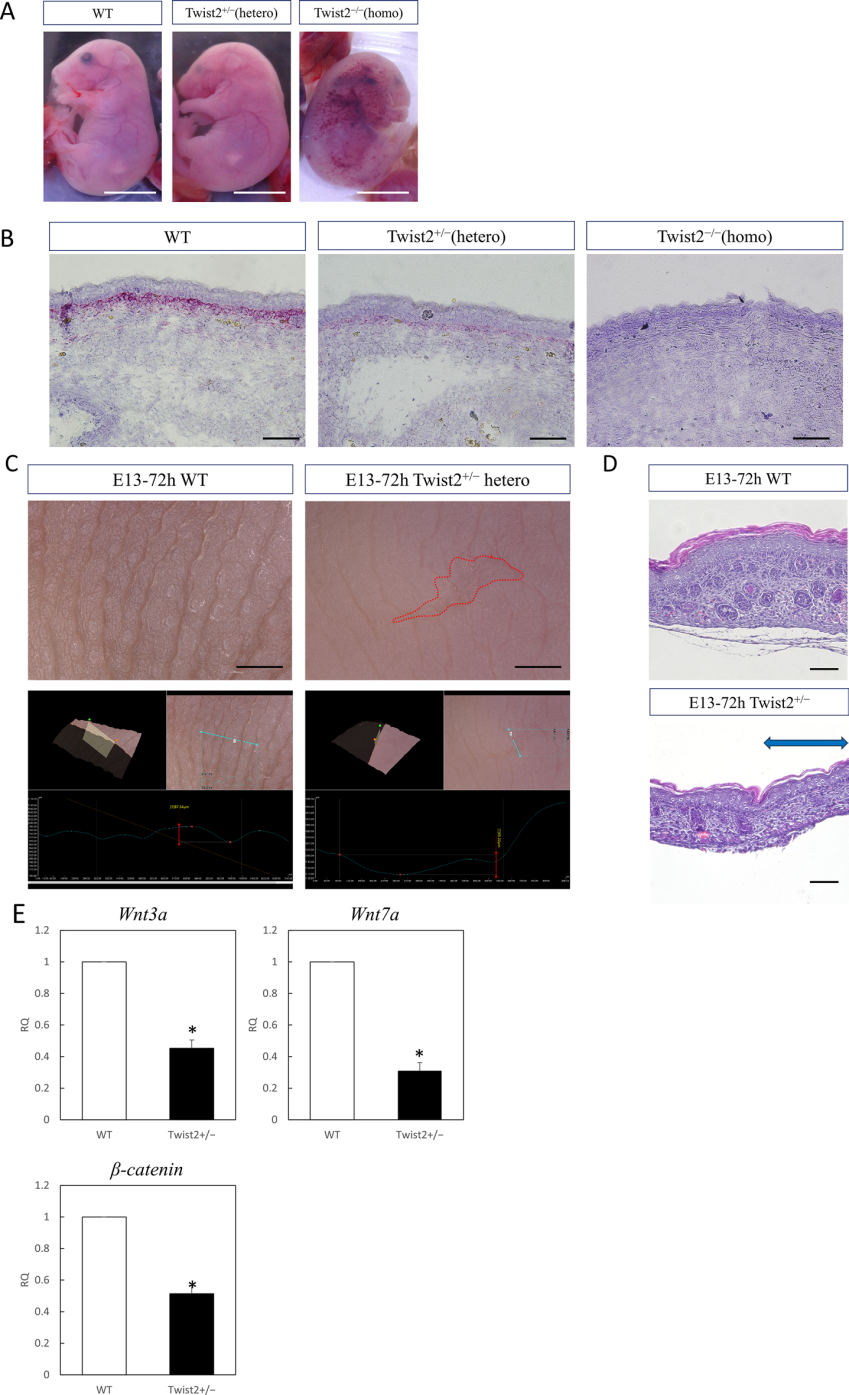
Wound healing in fetal Twist2 knockout mice. (**A**) Macrograph of Twist2 homozygous, heterozygous knockout, and wild type mice. Bar = 5 mm. Homozygotes showed congestion due to lymphedema. (**B**) Histology of normal skin of Twist2 homozygous, heterozygous knockout, and wild type E13 mice. Bar = 100 µm. No expression of Twist2 was observed in the dermis in the homozygous mice. (**C**) Macroscopic image of a wound of wild-type and Twist2 ± E13 mice at 72 h. Visible scarring was observed in Twist2-knockout mice. Bar = 500 µm. (**D**) Histological image of a wound on E13 after 72 h in wild type and Twist2 ± mice (H-E staining). Twist2-knockout mice showed no follicular regeneration in the wound area. Blue arrow: extent of wound. Bar = 100 µm. (**E**) qPCR analysis of hair induction-related gene expression in wound areas. RQ, relative quantification; qPCR, quantitative polymerase chain reaction. *P < 0.05.

Both hetero and homo fetuses survived post-placental surgery, with similar survival rates as wild type (WT) fetuses. Wound creation and retrieval were possible, but about half of the homo-operated individuals had congestive morphology with lymphangiogenesis imperfecta, and many showed severe edema (Fig. 3A). Histologically, the skin morphology was thinner in homo mice, and in situ hybridization showed no expression of Twist2 in these mice (Fig. 3B). Wounds were created in E13 Twist2-knockout mice, and changes in wound healing were examined. Wounds in WT mice regenerated without a visible scar in a normal pattern, whereas wounds in hetero mice had a visible scar and those on the body surface in homo mice were difficult to evaluate because of edema (Fig. 3C). WT mice regenerated all structures, including hair follicles, whereas Twist2-knockout mice did not regenerate hair follicles in the wound (Fig. 3D). In relation to this, the expression of *Wnt3a* (P = 0.00016), *Wnt7a* (P = 0.00024), and *β-catenin* (P = 0.0016) significantly decreased in the wounds of Twist2 hetero knockout mice compared to WT mice. (Fig. 3E).

### Twist2 expression is upregulated in 3D cultured fibroblasts

**Figure 4:**
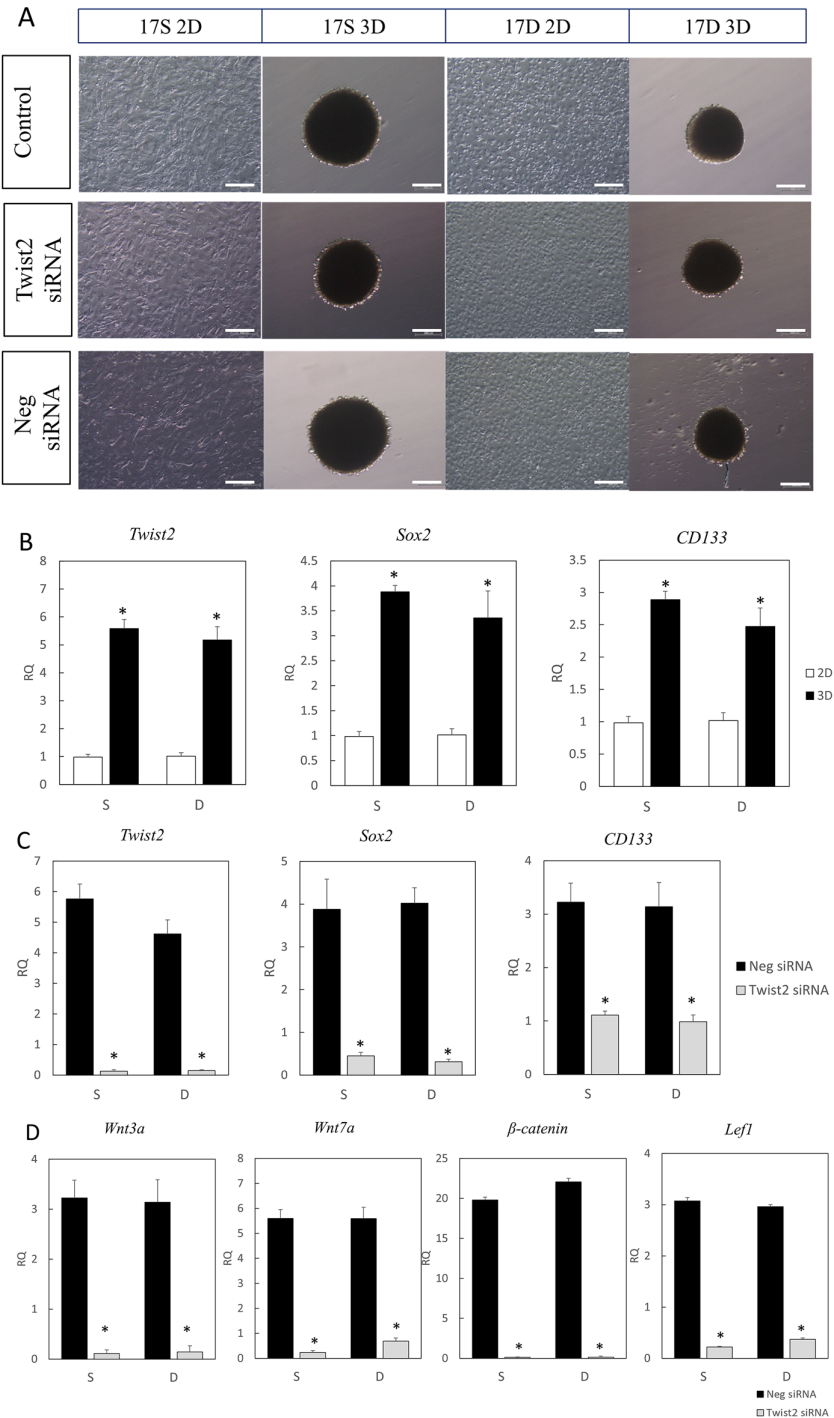
Two-dimensional (2D) and three-dimensional (3D) culture of dermal fibroblasts and observation of their ability to undifferentiate. (**A**) 2D and 3D culture of fibroblasts derived from the superficial and deep dermis layers on E17. S, superficial layer; D, deep layer. (**B**) qPCR analysis of the gene expression of Twist2 and undifferentiated markers. RQ, relative quantification. *P < 0.05. (**C**) qPCR analysis of the gene expression of undifferentiated markers after Twist2 knockdown. *P < 0.05. (**D**) qPCR analysis of hair induction-related gene expression. RQ, relative quantification; qPCR, quantitative polymerase chain reaction; Neg siRNA, negative control siRNA. *P < 0.05.

We previously reported that fetal dermal fibroblasts express undifferentiated markers, such as Sox2 and CD133, when cultured in nonadherent culture dishes and regain follicle-inducing ability once their expression is lost^22^. To investigate how Twist2 is involved in the expression of undifferentiated markers and in hair follicle formation, we performed in vitro knockdown of Twist2. We divided E17 fetal dermal fibroblasts into superficial (17S) and deep (17D) fibroblasts in relation to the dermal panniculus carnosus muscle. These cells were subjected to 2D or 3D culture using nonadherent culture dishes. No morphological changes were observed depending on the site of origin of the cells (Fig. 4A). However, reverse transcription-quantitative PCR (RT-qPCR) showed that the expression of *Twist2* (17S, P = 0.00056,17D, P = 0.0014) and the undifferentiated markers *Sox2* (17S, P = 0.0000056; 17D, P = 0.026) and *CD133* (17S, P = 0.00029; 17D, P = 0.018) was significantly upregulated in the 3D-cultured cells compared to the 2D-cultured cell (Fig. 4B). When Twist2 siRNA reagent was added to the 3D culture medium, *Twist2* expression was significantly knocked down (17S, P = 0.0075; 17D, P = 0.011), while *Sox2* (17S, P = 0.04; 17D, P = 0.0097) and *CD133* (17S, P = 0.028; 17D, P = 0.044) expression was significantly decreased compared with those when the negative control siRNA was used (Fig. 4C). In addition, the expression of *Wnt3a* (P = 0.0021), *Wnt7a* (P = 0.00019), *β-catenin* (P = 0.0016) and *Lef1* (P = 0.00031) significantly decreased in the Twist2 siRNA-treated group compared to the negative control siRNA-treated group (Fig. 4D).

### Knockdown of Twist2 reduces the migratory ability of dermal fibroblasts

**Figure 5:**
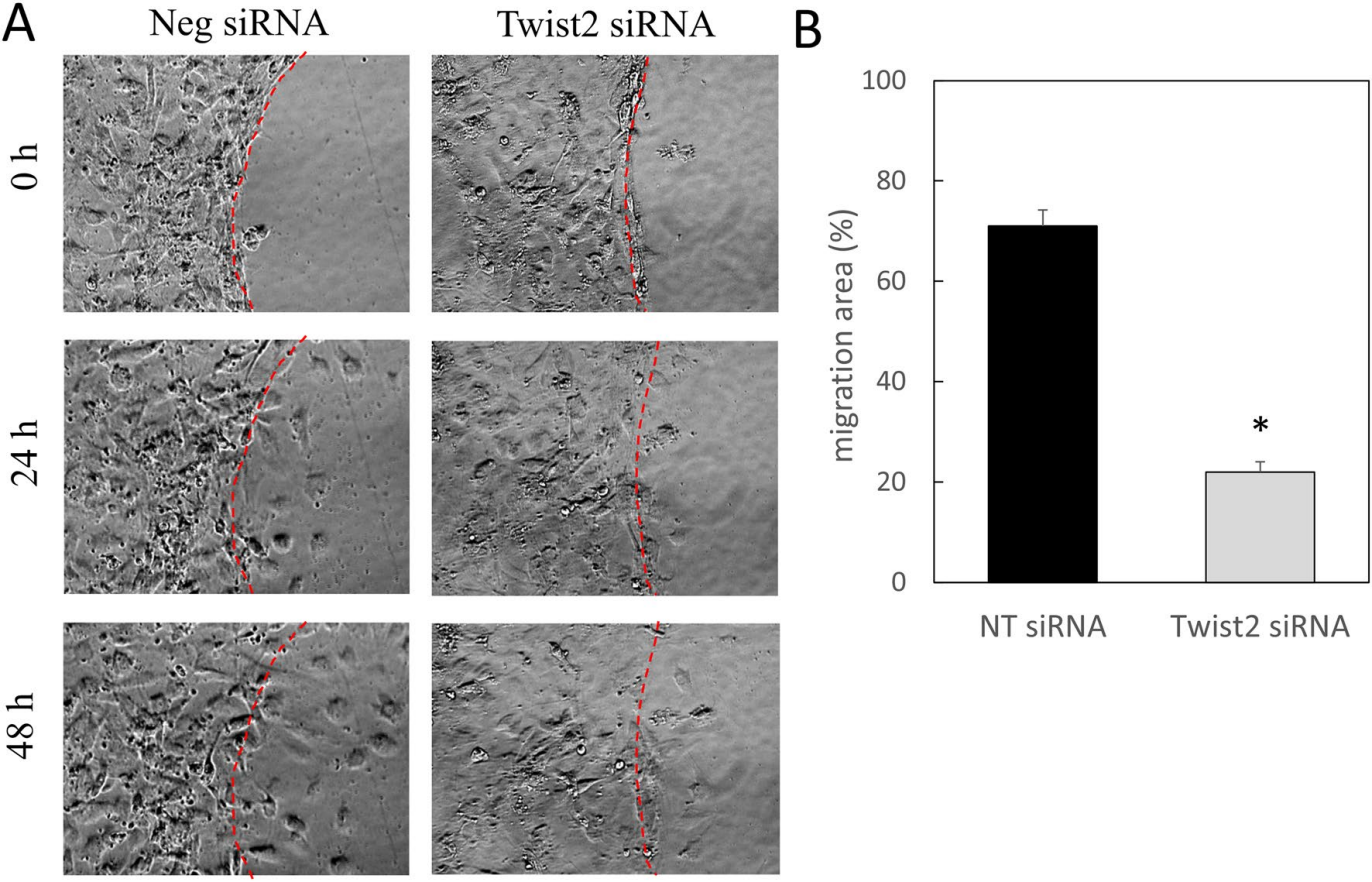
Alterations in the migratory capacity of deep dermal fibroblasts after Twist2 knockdown. (**A**) Scratch assay. Red dotted line: wound margin. (**B**) Epithelialization rate. Neg siRNA, negative control siRNA. *P < 0.05. All experiments were repeated three times. The area covered by migrated cells was measured as migration area in relation to the area of the gap created by scratching.

To further investigate the effect of Twist2 expression on wound healing in terms of cell migration, we performed a scratch assay and found that dermal fibroblasts with Twist2 knocked down had clearly reduced migratory ability (P = 0.0001; Fig. 5A,B). Thus, the expression of Twist2 in terms of cell migration ability also has an effect on wound healing.

## Discussion

Most adult mammalian tissues do not undergo regenerative healing, a process that reproduces functional tissue that is virtually indistinguishable from its original form^23^. Small skin wounds in mice result in the formation of scar tissue or fibrosis, which is primarily composed of ECM components, such as collagen and fibronectin, and lacks new accessory structures, such as hair follicles. This process is similar to that in wound skin in humans. However, the mediators that determine the balance between scar formation and regenerative skin wound healing remain unknown. In particular, in this not clear whether the scar prevents regeneration, whether scar cells inherently lack regenerative capacity, or whether there are simply no regenerative cues. Thus, there is a need to develop novel and efficient strategies for the treatment of this disorder, which requires a comprehensive understanding of the underlying cellular and molecular mechanisms.

We focused on Twist2, one of the factors involved in patterning and follicle formation, using an originally developed mouse embryonic wound healing model and compared it at the time points when wounds switch from regeneration to repair. The results showed that Twist2 expression was upregulated in the dermis of E13 wounds, which were completely regenerated, and was downregulated in wounds after E15. This expression of Twist2 was also located within the nucleus in E13 wound. Knockdown of Twist2 on E13 resulted in wound healing, leaving a visible mark, and inhibited regeneration. Interestingly, follicle formation was not observed in Twist2-knockdown wounds. Similarly, in twist2-knockout mice, wounds did not regenerate on E13 and follicle formation in the wound was also inhibited. To elucidate this mechanism, fibroblasts were made to acquire the ability to undifferentiate via 3D culture and were observed in vitro. The knockdown of Twist2 using siRNA or heterozygous deletion mutation of Twist2 reduced the expression of WNT/β-catenin signaling-related genes involved in follicle induction, downstream of Twist2, in the wound. The 3D-cultured dermal fibroblasts showed increased expression of undifferentiated markers as well as high expression of Twist2, and knockdown of Twist2 reduced the expression of these markers. Similar to the suppression of in vivo Twist2 expression, the expression of Wnt/β-catenin sub-signaling-related genes involved in hair follicle induction was downregulated by Twist2 knockdown in the 3D-cultured model. In terms of migration capacity, knockdown of Twist2 expression also reduced the migratory capacity of fibroblasts.

There have been only a few reports on the mechanism and role of Twist2 in skin wound healing. However, standard WNT signaling is known to be required for Twist2 expression in dermal fibroblasts during the embryonic stage when hair follicles are formed^13,24^. The WNT signaling pathway is an evolutionarily conserved intercellular communication system that is important for cell proliferation and differentiation in both development and tissue homeostasis^25^. In hair follicle morphogenesis, active WNT signaling in dermal fibroblasts via β-catenin and WNT co-transcription factors (such as Lef1, Tcf1, and Tcf4) is a prerequisite for dermal papilla layer commitment^26^. In addition, recent studies have shown that neonatal papillary fibroblasts still exhibit regenerative capacity supported by active WNT signaling at birth, even after the completion of fetal hair follicle morphogenesis^27^. Additionally, after birth, chromatin remodeling by Twist2 follows the fibroblast maturation pathway, spontaneous intracellular changes, resulting in the loss of regenerative capacity mediated by inactivation of the WNT signaling pathway^28^. It has been suggested that Twist2 expression decreases during postnatal maturation and that a major role of Twist2 is lineage-specific inactivation of WNT signaling, which occurs in a time-dependent manner after birth. Furthermore, transient loss of Twist2 expression in fibroblasts is thought to be necessary for maintaining high WNT signaling activity after the completion of dermal papillae differentiation^29^. The present study demonstrated that Twist2 is involved in complete skin regeneration and that its downregulation weakens Wnt/β-catenin signaling and induces a switch to healing that leaves a visible mark as in the adult wound type without hair follicle formation.

In terms of cell migration, fibroblasts in the dermis with Twist2 expression knocked down showed reduced migratory capacity. In relation to re-epithelialization, dermal recovery occurs through fibroblast migration and proliferation, and the response of fibroblasts during wound healing determines the outcome of tissue repair. In response to wounding, macrophages and fibroblasts secrete growth factors that result in further fibroblast migration and proliferation. Recently, transplantation and lineage tracing have identified two distinct dermal lineages that give rise to the upper and lower dermis, respectively^30^. Following wounding, both cell types are mobilized to the injured area,however, one population forms the lower dermis, whereas the other that gives rise to the upper dermis is mobilized during re-epithelialization and provides the environment for new follicle formation in the wounded area. In the present study, the results revealed altered expression of Twist2 in cells of the upper dermis and reduced cell migratory capacity upon Twist2 knockdown. Thus, the findings suggest that in wounds on E15 and later stages, reduced Twist2 expression in fibroblasts in the upper dermis leads to reduced migration and impaired follicle formation.

Despite these findings, a limitation of this study is the lack of analysis of the relationship between Twist2 expression and downstream Wnt signaling and target cells. Considering that the reticular dermis and subcutaneous fat also showed significant expansion in the postnatal period, it is possible that inactivation of the WNT signaling pathway by Twist2 is required for the postnatal differentiation of both upper and lower fibroblast lineage cells, and examination of the behavior of these cells in null mice is also needed^31^. The reduction in follicle induction by suppression of Twist2 expression observed in 3D-cultured cells will be further validated through observation of follicle formation in vivo by co-culturing epidermal cells and transplanting them into nude mice. Whether this phenomenon is applicable to mammals other than mice also needs to be confirmed.

The findings of the present study show that Twist2 expression is correlated with non-scarring skin regeneration and hair follicle formation during development. If this can be mimicked in the human skin and wound tissue, it may be useful for the development of scarless wound-healing and regenerative medicine.

## Methods

### Ethical consideration

The research protocol was reviewed and approved by the Institutional Animal Care and Use Committee of Keio University School of Medicine (approval number: 20170914). All experiments were conducted in accordance with the institutional guidelines for animal experiments at Keio University. This study is reported in accordance with the Animal Research: Reporting of In Vivo Experiments on Animals (ARRIVE) guidelines.

### Mouse

Jcl:ICR mice were obtained from Sankyo Laboratory Services, Inc. (Osaka, Japan). B6.129X1-Twist2^tm1.1(cre)Dor/J^ mice were purchased from Jackson Laboratories (#008,712, Bar Harbor, ME, USA). C57BL/6 J mice were used as WT mice. Heterozygous crosses were made and offspring were maintained. Phenotypes of the fetuses were confirmed using genotyping performed after fetal surgery and wound tissue was recovered.

### Fetal wounding procedure

Eight-week-old female ICR mice were used. Vaginal plugs were checked twice daily. The day when a plug was observed was designated as E0, and the fetuses were wounded on E13, E15, and E17. Surgeries were performed on five pregnant mice at each time point. Pregnant mice were anesthetized with 3% isoflurane, and the abdominal wall was incised to expose the uterus. Using an operating microscope, the myometrium and amniotic and yolk sacs were incised. Thereafter, using a pair of surgical microscissors, a full-layer incision approximately 2 mm in length was made in the lateral thoracic region of the fetus on E13. After wounding, the amnion and yolk sac were sutured with 9–0 nylon, whereas the myometrium was left open and unsutured. In particular, the fetus was returned to the abdominal cavity with the amnion and yolk sac covered but the myometrium uncovered, and the abdomen was closed; on E15 and E17, after fetal wounding, the myometrium was sutured with 9–0 nylon, uterus was returned to the abdominal cavity, and abdomen was closed. Just before the closure of both wounds, a uterine relaxant, ritodrine hydrochloride (FUJIFILM Wako Pure Chemical Co., Ltd., Osaka, Japan; 1 μg/g BW), was administered intraperitoneally. The peritoneum and skin were then sutured with 5–0 nylon thread. Maternal mice were euthanized via cervical dislocation, and fetuses were harvested 24 h after wounding. At each time point, wounds were made in at least four fetuses. Fetal skin was harvested and fixed in 4% paraformaldehyde for 24 h, and the fixed tissue was embedded in paraffin and stained.

### Immunohistochemistry

Paraffin-embedded specimens were sliced to 7-µm-thick sections and mounted on glass slides. After drying overnight at room temperature (15–25 °C) to allow the specimens to adhere to the slides, the paraffin was dissolved in a slide heater (ThermoBrite; Leica Biosystems, Nussloch, Germany) at 65 °C for 30 min immediately before use. The slides were then deparaffinized by changing xylene twice at room temperature (5 min each). The slides were transferred twice to 100% ethanol (3 min each), once to 95%, 70%, and 50% ethanol (3 min each), and rehydrated at room temperature. After antigen activation by heating, the samples were incubated with 2% goat serum in phosphate-buffered saline (PBS) for 30 min at room temperature to block nonspecific binding sites. The cells were then incubated with anti-Twist2 antibody (ab66031; Abcam, Cambridge, UK; diluted 1:100 in PBS) overnight at 4 °C. After washing three times with PBS, the cells were incubated with an anti-mouse HRPO-labeled rabbit IgG reagent, ImmPRESS (Vector Laboratories Inc., CA, USA; diluted 1:500 in PBS), for 1 h at room temperature. Signals were amplified using the avidin-biotinylated peroxidase complex method with the VECTASTAIN ABC kit (Vector Laboratories), and the sections were incubated in 20 mg/dL 3,3'-diaminobenzidine solution (FUJIFILM Wako Pure Chemical Co.) for 1–3 min. The sections were then washed once with running tap water for 5 min before counterstaining the nuclei for 6 s at room temperature with Gill’s hematoxylin solution (Merck Millipore, Billerica, MA, USA). Finally, the sections were washed with tap water for 5 min, dehydrated four times with ethanol (95%, 95%, 100%, and 100%; 5 min each), washed three times with xylene, and sealed with Mount Quick Sealant (Takara Bio, Shiga., Japan). The slides were observed under an integrated stereo microscope (BZ-X800; KEYENCE, Osaka, Japan). The experiment was repeated three times.

### In Situ hybridization

In situ hybridization analysis was performed using the QuantiGene ViewRNA ISH Tissue Assay (Thermo Fisher Scientific, Waltham, MA, USA) according to the manufacturer’s instructions. Briefly, the paraffin-embedded specimens were dried at 60 °C for 60 min, and paraffin was removed using Histo Clear (National Diagnostics, Atlanta, GA, USA) and 100% ethanol with ImmEdge Pen (Vector Laboratories Inc.). After washing with PBS twice, the tissue was fixed in 10% neutral-buffered formalin solution for 5 min and washed again with PBS. The target probe was diluted 50-fold in probe set diluent QF solution warmed to 40 °C and incubated at 40 °C for 3 h. After washing with wash buffer three times, the preamplifier solution was incubated at 40 °C for 25 min. The preamplifier solution was washed with wash buffer three times and then incubated at 40 °C for 15 min. The AP enhancer solution was decanted, and the samples were incubated in a solution of Fast Red Tablet dissolved in Naphthol buffer at room temperature for 5 min. After decanting the AP enhancer solution, the samples were incubated in a solution of Fast Red Tablet dissolved in Naphthol buffer at 40 °C for 30 min. After washing with PBS twice, nuclear staining was performed with Gill’s hematoxylin solution, and the cells were washed with water three times. The probe used was Twist2 (VB1-14748-VT).

### LMD, RNA isolation, and reverse transcription

LMD was performed using PALM MicroBeam (Carl Zeiss, Germany). The manufacturer’s recommended slides and collection tubes (AdhesiveCap 500 opaque; Carl Zeiss) were set up, and the tissue was carefully cut after adjusting the aperture and intensity using a 20× magnification objective lens. The tube caps were filled with Buffer RLT (RNeasy Micro Kit; Qiagen, Germany) with β-mercaptoethanol to allow the separation of intact RNA. Total RNA was extracted from cells or skin tissue using a monophasic solution of phenol and guanidine isothiocyanate (ISOGEN; Nippon Gene, Tokyo, Japan) according to the manufacturer’s instructions. Total RNA was mixed with a random primer, reverse transcriptase, and dNTP mixture (Takara Bio). Reverse transcription was performed on a T100TM thermal cycler (Bio-Rad Laboratories, Inc., Hercules, CA, USA) at 25 °C for 5 min, 55 °C for 10 min, and 80 °C for 10 min to heat-inactivate the reverse transcriptase and produce cDNA.

### qPCR

qPCR was performed using the Applied Biosystems 7500 Fast Real-Time PCR System (Thermo Fisher Scientific). A total of 40 cycles were performed, and the fluorescence of each sample was measured at the end of each cycle. The PCR conditions used were as follows: 95 °C for 3 s (denaturation) and 60 °C for 30 s (annealing and extension). In the subsequent melting curve analysis phase, the temperature was increased from 60 °C to 95 °C and fluorescence was measured continuously. Twist2 (Mm00492147_m1), Sox2 (Mm03053810_s1), Wnt3a (Mm00437337_m1), Wnt7a (Mm00437356_m1), Ctnb1 (β-catenin) (Mm00483039_m1), Lef1 (Mm00550265_m1), and prom1 (CD133) (Mm00477115_m1) were used as primers (all from Thermo Fisher Scientific). The PCR master mix (cat. 4,352,042; Applied Biosystems, Foster City, CA, USA) was used according to the manufacturer’s instructions, and *ACTB* (Mm02619580_g1) was used as a control gene for normalization according to the manufacturer’s instructions. Gene expression levels at normal sites were used as baseline, and fold-change values were determined using the 2-ΔΔCt method.

### siRNA experiment for mouse fetuses

E13 mice were subjected to normal fetal surgery, and siRNA reagents were injected into the amniotic fluid as previously reported^32^. The concentration of Twist2 siRNA reagent (160,520 and 61,236,Thermo Fisher Scientific) used was 1.5 mg/mL, approximately 100 µM concentration. Equal amounts of complex buffer (1,377,501, Thermo Fisher Scientific) and siRNA were mixed with a double volume of invivofec-tamine (1,377,501, Thermo Fisher Scientific) to prepare the reagent. Negative siRNA (Silencer™ Negative Control No. 1 siRNA; Thermo Fisher Scientific) was used as a negative control. The prepared reagents were then incubated at 50 °C for 30 min, placed in a Float-A-Lyzer dialyzer, and dialyzed with 1 L of PBS (pH 7.4). The final concentration of siRNA preparation reagent (approximately 0.35 mg/mL) was administered in approximately 40 µL amniotic fluid per fetal mouse. Tissues were harvested 72 h later, and the recovered tissues were embedded in paraffin. Specimens were sliced to 7-µm-thick sections and mounted on glass slides, and hematoxylin and eosin-stained tissues were observed.

### 2D and 3D culture of fetal mouse dermal fibroblasts

E17 fetal mouse skin was divided under a microscope into the superficial (S) and deep (D) layer of the dermis. Fibroblasts were established from these tissues, and those at passage 5 were used as mesenchymal cells. They were cultured as previously described^22^ in two-dimensional culture using a regular adherent culture dish (Nunclon Delta,Thermo Fisher Scientific) and in three-dimensional culture using a dish that was made non-adherent by coating it with 1% agarose gel. Next, 10% fetal bovine serum and Dulbecco’s modified Eagle medium with 1% penicillin–streptomycin were used for each culture. Cells were grown at 37 °C and 5% CO_2_, and the medium was changed twice a week. Three-week-old cultures were then incubated with Lipofectamine 2000 (11,668–019; Life Technologies, Invitrogen, Germany) and then transfected with Twist2 siRNA (160,520 and 61,236, Silencer™ siRNA; Thermo Fisher Scientific) or non-targeted siRNA (4,390,843, Silencer™ Select Negative Control No. 1 siRNA; Thermo Fisher Scientific) via injection into the culture medium. After 72 h, RNA was collected from the cells using ISOGEN (Nippon Gene), and specific gene knockdown was evaluated via RT-qPCR.

### Scratch assay

Knockdown of Twist2 in fibroblasts in the dermis layer was performed in the same manner as in 3D culture. The cells were treated with 10 µg/mL mitomycin C (NACALAI TESQUE, INC., Kyoto, Japan) for 3 h at 37 °C to exclude proliferative effects prior to the assay. The cells were grown to confluence on a plastic dish and scratched using a 500-µm pipette tip. The cells were grown in Dulbecco’s modified Eagle medium at 37 °C under 5% CO_2_ and 95% relative humidity for up to 24 h. Migration of cells from the edges of the scratches was visualized under a microscope (BZ-X800; KEYENCE). The area covered by migrating cells was measured as migration area in relation to the area of the gap by scratching.

### Statistical analysis

Mann–Whitney U test was performed using the Statistica software version 9.0 (StatSoft, Tulsa, OK) to determine the significance of differences in gene expression. The results of descriptive statistics are presented as mean ± standard deviation. Statistical significance was set at P < 0.05. Each experiment was performed in triplicate.

## Data Availability

The data presented in this study are available on request from the corresponding author.
